# Massively Parallel Signature Sequencing and Bioinformatics Analysis Identifies Up-Regulation of TGFBI and SOX4 in Human Glioblastoma

**DOI:** 10.1371/journal.pone.0010210

**Published:** 2010-04-19

**Authors:** Biaoyang Lin, Anup Madan, Jae-Geun Yoon, Xuefeng Fang, Xiaowei Yan, Taek-Kyun Kim, Daehee Hwang, Leroy Hood, Gregory Foltz

**Affiliations:** 1 Swedish Neuroscience Institute, Swedish Medical Center, Seattle, Washington, United States of America; 2 Department of Urology, University of Washington, Seattle, Washington, United States of America; 3 Zhejiang-California International NanoSystems Institute, Zhejiang University, Hangzhou, Zhejiang, China; 4 The Institute for Systems Biology, Seattle, Washington, United States of America; 5 Seattle Molecular Diagnostics Research Institute, Bellevue, Washington, United States of America; 6 I-Bio Program and Department of Chemical Engineering, Pohang University of Science and Technology, Pohang, Kyungbuk, Republic of Korea; Institute of Cancer Research, United Kingdom

## Abstract

**Background:**

A comprehensive network-based understanding of molecular pathways abnormally altered in glioblastoma multiforme (GBM) is essential for developing effective therapeutic approaches for this deadly disease.

**Methodology/Principal Findings:**

Applying a next generation sequencing technology, massively parallel signature sequencing (MPSS), we identified a total of 4535 genes that are differentially expressed between normal brain and GBM tissue. The expression changes of three up-regulated genes, CHI3L1, CHI3L2, and FOXM1, and two down-regulated genes, neurogranin and L1CAM, were confirmed by quantitative PCR. Pathway analysis revealed that TGF- β pathway related genes were significantly up-regulated in GBM tumor samples. An integrative pathway analysis of the TGF β signaling network identified two alternative TGF−β signaling pathways mediated by SOX4 (sex determining region Y-box 4) and TGFBI (Transforming growth factor beta induced). Quantitative RT-PCR and immunohistochemistry staining demonstrated that SOX4 and TGFBI expression is elevated in GBM tissues compared with normal brain tissues at both the RNA and protein levels. *In vitro* functional studies confirmed that TGFBI and SOX4 expression is increased by TGF- β stimulation and decreased by a specific inhibitor of TGF- β receptor 1 kinase.

**Conclusions/Significance:**

Our MPSS database for GBM and normal brain tissues provides a useful resource for the scientific community. The identification of non-SMAD mediated TGF−β signaling pathways acting through SOX4 and TGFBI (GENE ID:7045) in GBM indicates that these alternative pathways should be considered, in addition to the canonical SMAD mediated pathway, in the development of new therapeutic strategies targeting TGF−β signaling in GBM. Finally, the construction of an extended TGF- β signaling network with overlaid gene expression changes between GBM and normal brain extends our understanding of the biology of GBM.

## Introduction

Glioblastoma multiforme (GBM), the most common type of primary brain cancer, is currently incurable and uniformly fatal. A comprehensive understanding of molecular pathways underlying GBM behavior would enable the development of targeted therapeutic approaches.

In the past several years, DNA microarrays have been used to identify differential gene expression among different grades of brain tumors [1], for tumor classification [2]–[4], prognosis [5], [6], and screening for epigenetic changes [7], [8]. Despite these advances, current DNA microarray technology has limited detection sensitivity and dynamic range [9] which limits its ability to detect changes in gene expression at low levels of expression. As a large number of genes fall into this class of low abundance expression [10], this lack of sensitivity potentially compromises current efforts to gain a complete picture of molecular pathways underlying GBM. To gain a more comprehensive and system-wide understanding of molecular pathways in GBM, more sensitive gene expression profiling technology is needed.

Massively parallel sequencing of expressed sequenced tags (also named massively parallel signature sequencing, MPSS) is a more sensitive technology in reliably detecting low expression transcripts [10]–[12] and has been shown to complement current DNA microarray technologies [13]. We have therefore used this technology to help us gain a more complete picture of the molecular events and networks perturbed in GBM. We applied MPSS technology to compare the expression profiling of a pool of five normal brain tissues to a pool of five GBM tissues. With the MPSS technology, we were able to identify differential expression of low abundance genes. We found activation of two alternative non-SMAD mediated TGF–β signaling subnetworks that act through SOX4 (sex determining region Y-box 4) and TGFBI (transforming growth factor β induced transcript). *In vitro* studies confirmed that both SOX4 and TFBI are induced by TGF-β and inhibited by a specific inhibitor of the TGF- β receptor 1 kinase.

## Results

### MPSS analysis of normal brain and GBM tissues

We sequenced a total of 1,479,906 and 1,521,666 tags respectively from a pool of five normal brain tissues and a pool of five GBM tissues. We identified 22,640 MPSS tags that have significantly expressed tags (>3 tpm in at least one pool), representing the combined transcriptome of the normal and GBM tumor tissues (Table S1). 96% of the tags could be mapped to the human genome (hg18), of which 10.1% were repeats or mapped to multiple genomic locations. The remaining 84.9% of the tags uniquely mapped to the human genome with 9.6% of these tags mapping to unannotated regions.

The majority of MPSS tags (75.3%) mapped to previously characterized genes (with at least one EST sequence as evidence). MPSS tags are categorized into different classes based on their mapped location and orientation to known cDNAs (Refseq or ESTs)[10], [12]. We limited ourselves to the analysis of MPSS tags that belong to classes1 through 5 as these are considered to have more reliable annotation[10], [12]. We identified a total of 13,606 class 1–5 MPSS tags of which 12,208 corresponded to 8,518 defined genes (i.e. with Entrez Gene ID or Unigene IDs). This represented an average of 1.43 MPSS tags per gene (Table S2), suggesting alternative polyadenylation of some genes as MPSS technology (described in detail in the method section) captures the last GATC tag closest to the poly A tail of genes [11]. In addition, 1,395 tags corresponded to unannotated genes or ESTs which may represent novel transcripts or novel isoforms of known genes. The MPSS data have been submitted to the GEO database with the accession number GPL8198.

**Figure 1:**
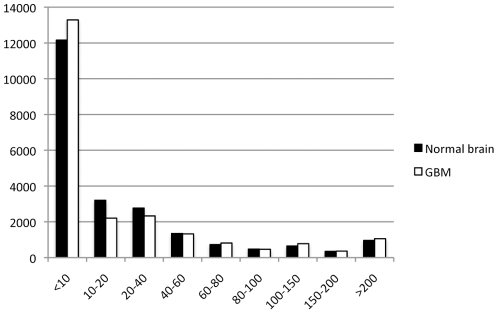
Bar chart showing the frequencies of MPSS tags expressed at different levels (bins at 1–10 tpm, 11–20 tpm etc.). Y-axis, numbers of MPSS tags; X-axis, bins of expression levels. About half of the transcripts were expressed at low levels (<10 tpm).

We found that MPSS was able to detect many transcript expressed at low levels. Figure 1 shows the distribution of MPSS tags at different abundance levels in transcripts per million (tpm). We observed that about 68% of transcripts were expressed at less than 20 tpm in normal brain tissues or GBM tissues. This illustrates the sensitivity of next generation sequencing technology in identifying lowly expressed transcripts.

To identify differentially expressed genes, we used the *Z*-test [14], [15] to compare gene expression between normal and GBM tumor tissue. For multiple testing corrections, we computed the false discovery rate (FDR) for each tag using statistical hypothesis testing involving Storey's method [15] (see Materials and Methods for detail). Using a FDR cutoff of 0.1, we identified 3,352 tags that show significantly differential expression (Table S3). Among these, 1,614 tags (1,391 genes) are up regulated in GBM compared to normal brain (Table S3) and 1,738 tags (1,451 genes) are down regulated in GBM compared to normal brain (Table S4).

### Confirmation of MPSS data by real-time RT PCR and identification of putative biomarkers for GBM

**Figure 2:**
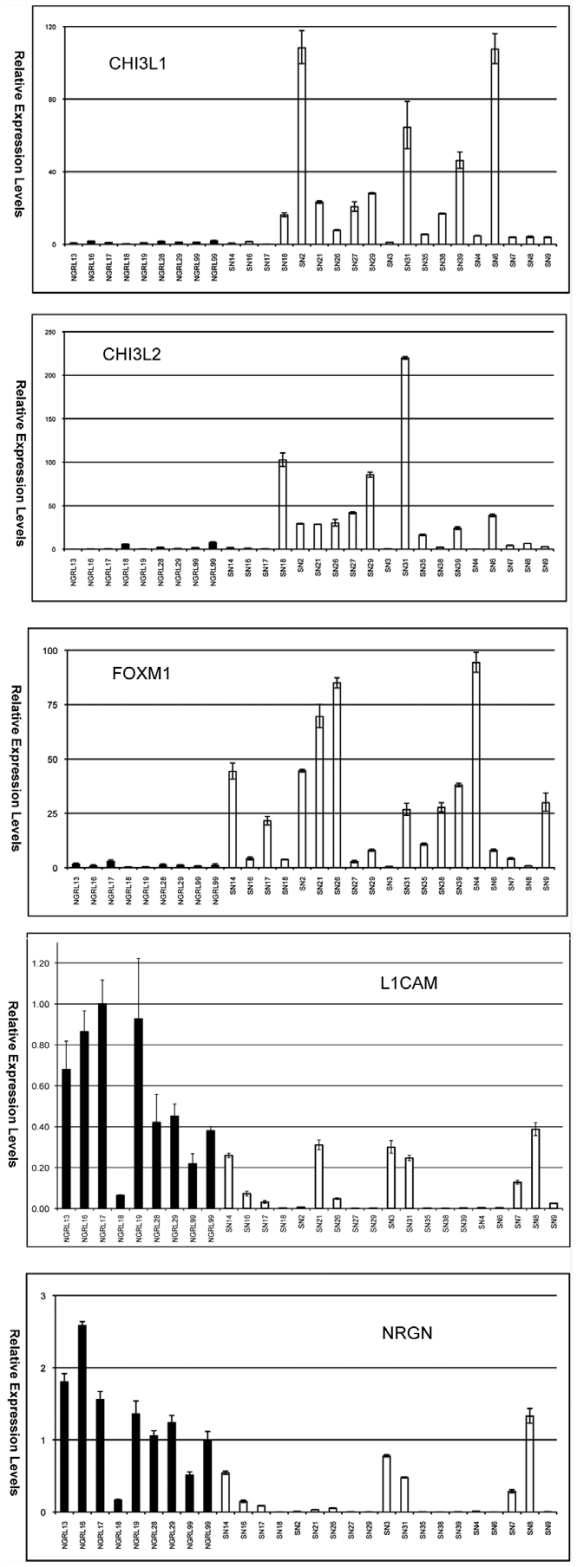
Bar charts showing the quantitative RT-PCR results of CHI3L1, CHI3L2, FOXM1, NRGN and L1CAM on a panel of 19 individual brain tumor samples (SN series) and 9 individual normal brain tissue samples (NGRL series). Black bars, NGRL series (normal) samples; white, SN series (GBM) samples. Y-axis indicates relative expression levels and X-axis indicates individual samples. Three replicate PCR were performed and the standard errors of the mean were indicated by error bars.

To confirm differential gene expression in individual samples, we randomly picked two up-regulated genes, FOXM1 (forkhead box M1) and CHI3L1 (chitinase 3-like 1), and two down regulated genes, NRGN (neurogranin) and L1CAM (L1 cell adhesion molecule), and evaluated them in a panel of 19 individual brain tumor samples and 9 individual normal brain tissues. The expression values of these genes in the MPSS data from pooled samples are shown in Table S5. Using real time quantitative PCR, we confirmed that CHI3L1 and FOXM1 were significantly up regulated (P<0.05, T test) and confirmed that NRGN and L1CAM were significantly down regulated (P<0.05, T test) in GBM compared to normal brain tissues (Figure 2).

CHI3L1 (chitinase 3-like 1, also named YKL-40) was identified previously as a differentially expressed gene in GBM compared with normal brain tissues [16]. CHI3L1 is a member of mammalian chitinase-like proteins [17]. Interestingly, there is another member of the mammalian chitinase-like proteins, CHI3L2 (YKL39) [18]. We were intrigued as to whether CHI3L2 is also differentially expressed. We found the CHI3L2 was also over expressed in GBM tissues compared to normal brain tissues (Table S5) and confirmed it by real time quantitative PCR (Figure 2).

### Identification of enriched gene sets of differentially expressed genes in GBM compared to normal brain tissues

To understand which Gene Ontology terms are enriched with GBM related genes, we performed gominer (http://discover.nci.nih.gov/gominer/) analysis. We found that genes over-expressed in normal brain tissues compared to GBM are enriched for GO terms related to normal brain cellular functions such as GO:0007268 synaptic transmission, GO:0019226 transmission of nerve impulse, GO:0007268 synaptic transmission, GO:0007399 nervous system development, GO:0048699 generation of neurons, and GO:0050877 neurological system process.

However, GO analysis of genes over-expressed in GBM compared to normal brain tissues revealed enrichment of general GO terms in basic cellular metabolic and biosynthesis processes such GO:0031328 positive regulation of cellular biosynthetic process, GO:0045941 positive regulation of transcription, GO:0010467 gene expression.

As this initial analysis only identified general changes related to normal brain functions in normal tissues or increased cell metabolism in GBM, we decided to further refine the gene sets enriched in the differentially expressed genes between GBM and normal brain tissues using Gene Set Enrichment Analysis (GSEA) [19]. After GSEA, we found that there were 92 gene sets (Table S6) significantly enriched in GBM, and 24 gene sets (Table S7) significantly enriched in normal brain tissues. For this analysis, we used a threshold FDR q value <0.25 which is the standard FDR rate recommended for GSEA analysis [19]. The top enriched gene sets with FDR q value <0.05 is shown in Table 1.

**Table 1:** Top enriched gene sets in GBM and normal brain tissues (FDR<0.05).

NAME	SIZE	ES	NES	NOM p-val	FDR q-val
**Enriched gene sets in GBM**					
STEMCELL_NEURAL_UP	370	−0.2	−3.6	0.0000	0.0000
CARIES_PULP_UP	40	−0.4	−3.3	0.0000	0.0000
TGFBETA_ALL_UP	30	−0.4	−2.9	0.0000	0.0014
HSA04115_P53_SIGNALING_PATHWAY	16	−0.6	−2.8	0.0000	0.0014
CMV_24HRS_DN	23	−0.5	−2.7	0.0000	0.0028
LEI_MYB_REGULATED_GENES	81	−0.3	−2.6	0.0000	0.0028
BREAST_CANCER_ESTROGEN_SIGNALING	24	−0.4	−2.5	0.0000	0.0035
CMV_ALL_DN	31	−0.4	−2.5	0.0000	0.0031
TGFBETA_EARLY_UP	21	−0.5	−2.5	0.0000	0.0035
CELL_CYCLE_KEGG	18	−0.5	−2.5	0.0000	0.0060
VHL_NORMAL_UP	115	−0.2	−2.4	0.0000	0.0067
G1_TO_S_CELL_CYCLE_REACTOME	15	−0.5	−2.4	0.0000	0.0087
CELL_CYCLE	17	−0.5	−2.4	0.0000	0.0085
GAY_YY1_DN	53	−0.3	−2.3	0.0000	0.0116
HSA05222_SMALL_CELL_LUNG_CANCER	16	−0.5	−2.3	0.0020	0.0148
CARIES_PULP_HIGH_UP	16	−0.5	−2.3	0.0020	0.0231
ALZHEIMERS_DISEASE_UP	300	−0.1	−2.2	0.0000	0.0228
HUMAN_CD34_ENRICHED_TRANSCRIPTION_FACTORS	37	−0.3	−2.2	0.0000	0.0229
LEE_MYC_E2F1_UP	15	−0.5	−2.2	0.0000	0.0240
LEE_E2F1_UP	16	−0.5	−2.2	0.0040	0.0250
KENNY_WNT_UP	15	−0.5	−2.2	0.0000	0.0272
STEMCELL_EMBRYONIC_UP	210	−0.1	−2.2	0.0000	0.0308
CANCER_NEOPLASTIC_META_UP	25	−0.4	−2.1	0.0000	0.0319
IDX_TSA_UP_CLUSTER3	18	−0.4	−2.1	0.0042	0.0399
SERUM_FIBROBLAST_CELLCYCLE	22	−0.4	−2.1	0.0038	0.0424
TNFALPHA_ALL_UP	21	−0.4	−2.1	0.0000	0.0455
LEE_TCELLS10_UP	19	−0.4	−2.1	0.0019	0.0463
ESR_FIBROBLAST_UP	18	−0.4	−2.0	0.0022	0.0488
LEE_TCELLS8_UP	19	−0.4	−2.0	0.0000	0.0477
LEE_TCELLS1_UP	19	−0.4	−2.0	0.0060	0.0482
AGEING_KIDNEY_SPECIFIC_UP	36	−0.3	−2.0	0.0040	0.0487
UVB_NHEK1_DN	43	−0.3	−2.0	0.0040	0.0489
LEE_TCELLS2_UP	202	−0.1	−2.0	0.0020	0.0477
SHEPARD_BMYB_MORPHOLINO_DN	38	−0.3	−2.0	0.0082	0.0469
HSA03010_RIBOSOME	34	−0.3	−2.0	0.0021	0.0458
WIELAND_HEPATITIS_B_INDUCED	22	−0.4	−2.0	0.0000	0.0448
RAS_ONCOGENIC_SIGNATURE	47	−0.3	−2.0	0.0039	0.0468
HSA04512_ECM_RECEPTOR_INTERACTION	18	−0.4	−2.0	0.0000	0.0476
VERHAAK_AML_NPM1_MUT_VS_WT_UP	34	−0.3	−2.0	0.0141	0.0485
BRCA_ER_NEG	169	−0.1	−2.0	0.0039	0.0473
JISON_SICKLECELL_DIFF	108	−0.2	−2.0	0.0021	0.0482
DNA_DAMAGE_SIGNALING	19	−0.4	−2.0	0.0062	0.0490
**Enriched gene sets in normal brain tissues**					
ALZHEIMERS_DISEASE_DN	342	0.3	5.8	0.0000	0.0000
CALCIUM_REGULATION_IN_CARDIAC_CELLS	39	0.4	2.9	0.0000	0.0000
ASTON_DEPRESSION_DN	54	0.3	2.4	0.0000	0.0234
DFOSB_BRAIN_8WKS_UP	17	0.5	2.4	0.0000	0.0187
AGEING_BRAIN_DN	43	0.3	2.2	0.0042	0.0467

Of note, the top enriched gene sets include two TGF−β related gene sets: the TGFBETA ALL UP and the TGFBETA EARLY UP, ranked number 3 and 9 with FDR q values of 0.001 and 0.004 respectively (Table 1). The TGFBETA EARLY UP gene set contains 58 genes found to be up-regulated by TGF-beta treatment of skin fibroblasts at a 30 minute time point while the TGFBETA_ALL_UP gene set contains 90 genes up-regulated by TGF-beta treatment of skin fibroblasts at multiple early and late time points (30, 60, 120, 240 minutes) [20]. Although the canonical TGF−β signaling pathway gene set (defined by KEGG pathway classification) as a whole was not significantly enriched by GSEA analysis (FDR q value of 0.97), a visual inspection of the overlaid gene expression changes onto the canonical TGF−β signaling pathway revealed several genes including SMAD2 (FDR  = 0.038) and SMAD7 (FDR  = 0.007) which were over expressed in GBM tissues compared to normal brain tissues (Figure S1). Activated SMAD2 is a key transducer of TGF−β signaling that binds to SMAD4 and is translocated into the nucleus to initiate transcription of downstream target genes. Furthermore, in addition to TGF−β signaling itself, other members of the TGF−β superfamily such as bone morphogenesis protein 1 (BMP1) and genes activated through TGF−β superfamily members activin and nodal, such as activinRIII and nodalRII, are also upregulated in GBM (Figure S1). These pathways all act through SMAD proteins and are considered SMAD-mediated TGF−β signaling pathways.

### The TGF–β network in GBM

Pathway databases such as KEGG and other commercial and non-commercial sources (e.g. Biocarta) typically only include a few genes such as those listed in the canonical TGF–β signaling pathway. GSEA, on the other hand, only provides a list of genes without network relationships. To overcome the limited gene number in canonical pathway maps and the lack of network links in the enriched GSEA gene sets, we constructed a TGF–β network which could then be overlaid with our gene expression data. The overlaid network was displayed in the network browser Cytoscape which allowed identification of key nodal changes in the network. As previously described, we chose TGF–β to demonstrate the utility of this approach as we had identified two TGF–β related gene sets as the top ranking gene sets (Table 1) and observed several significant gene expression changes in the canonical TGF–β pathway. For analysis, we first constructed a TGF–β interaction network by compiling all existing public information with no new edges added from our data. The resulting network was then overlaid with expression ratios (red colored nodes indicate over-expression and blue colored nodes indicate under-expression) from our MPSS analysis of GBM tissues and normal brain tissues. This allowed for the identification of key changes in the network when comparing normal brain tissue and GBM.

We started by assembling a list of known TGF–β regulated genes by integrating three kinds of data type: protein-protein interactions, microarrays and ChIP-Chip analysis. We first identified 64 proteins that interact with TGF–β using the Michigan Molecular Interaction tool MiMI (http://portal.ncibi.org/gateway/mimi.html). We then compiled a list of genes regulated by TGF–β from various microarray studies. Lesne et al. used a brain cDNA microarray to identify TGF–β regulated genes in TGF–β 1 treated cultures of cortical neurons and astrocytes in the mouse [21]. We retrieved the human homologues for these differentially expressed genes. We also included differentially expressed genes identified by microarray studies for TGF–β responsive genes in the GEO microarray database, which includes TGF–β regulated genes in acute myelogenous leukemia cells (GSE1805 in the GEO database)[22], in lung carcinoma (A549) (GSE7436 in the GEO database), in immortalized lung epithelial (HPL1D) cells [23], in MDA-MB-468 Smad4 positive/negative clones treated with TGF–β (GSE2567), in human HP75 pituitary cell line [24], in breast cancer cells (GSE5265) [25] and in two glioma cell lines U373MG and U87MG [26]. Of interest, Scharer et al. recently used ChIP-chip technology to identify 23 direct targets of SOX4 in prostate cancer cells [27]. We added these 23 directly targeted transcription factors to the SOX4 subnetwork [27]. In the end, we identified a list of 1,678 genes which are either regulated by TGF–β or exhibit potential as protein-protein interaction partners (Table S8).

As the TGF–β regulated genes that we compiled from the microarray data above does not contain interaction information, we used Cytoscape with the MiMI plugin [28] to identify protein-protein interactions. Cytoscape is a widely used open source software tool for displaying interaction of molecules [29]. The MiMi plugin for Cytoscape (http://mimiplugin.ncibi.org/) was developed to facilitate access to the molecular interaction data assembled in MiMI that contains integrated data from multiple well-known protein interaction databases using an intelligent deep-merging approach [30]. The MiMi plugin retrieves molecular interactions and interaction attributes from MiMI and displays the interaction networks and attributes using Cytoscape [28]. Using this newly defined TGF–β interaction network, we asked the question how many genes in this network correspond to differentially expressed genes (FDR <0.1) that we had identified between normal brain tissue and GBM tumors. Using this approach, we identified a final list of 420 presumed TGF–β regulated genes that are differentially expressed in GBM (Table S8).

**Figure 3:**
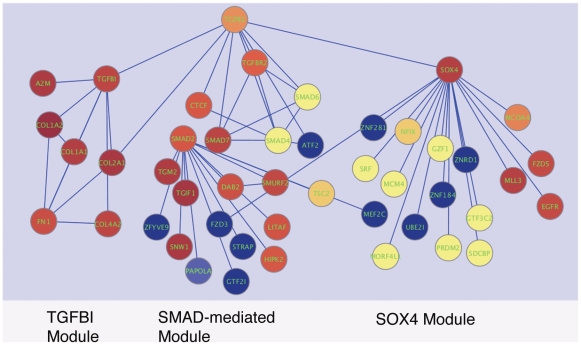
The SMAD2 mediated module (canonical TGF beta pathway), SOX4 module and TGFBI (GENE ID:7045) module of the TGF−β network. The expression ratios of GBM tissues to normal brain tissues are overlaid onto the network. Red color indicates over expression, yellow color indicates no changes, and blue color indicates under expression.

Exploring the resulting network displayed by Cytoscape revealed a subnetwork centered around TGF–β This subnetwork captured the canonical TGF–β signaling molecules (Figure 3). Of note, additional interesting genes appeared in the network, of which two interested us most: SOX4 (sex determining region Y-box 4) and TGFBI (transforming growth factor beta 1 induced transcript) (Figure 3). As SOX4 and TGFBI are two key genes in the network and neither of these genes had been previously described in GBM, we decided to further analyze the expression of SOX4 and TGFBI in GBM.

### SOX4 and TGFBI are TGF–β regulated genes over expressed in GBM

**Figure 4:**
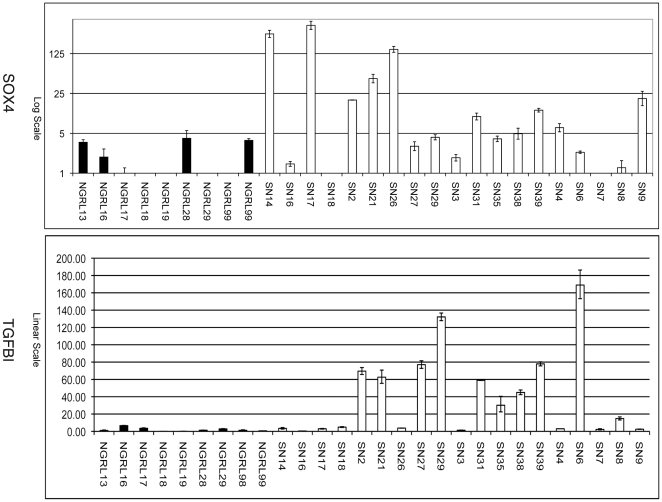
Bar charts showing the quantitative RT-PCR results of SOX4 and TGFBI (GENE ID:7045) on a panel of 19 individual brain tumor samples (SN series) and 9 individual normal brain tissue samples (NGRL series). Black bars, NGRL series (normal) samples; white, SN series (GBM) samples. Y-axis indicates relative expression levels and X-axis indicates individual samples. Three replicate PCR were performed and the standard errors of the mean were indicated by error bars. Both TGFBI (GENE ID:7045) and SOX4 were differentially expressed with increased expression in GBM tissues compared to normal brain (P<0.01 for both TGFBI (GENE ID:7045) and SOX4, T-test, two-tailed distribution, unequal variance). Please note that the Y-axis for SOX4 is in log scale in order to show the full extend of SOX4 expression in the samples.

There are three MPSS tags for SOX4 and two MPSS tags for TGFBI sequenced in our MPSS data (Table S5). The three tags for SOX4, although belonging to different classes, showed significantly higher expression in GBM comparing with normal brain tissues. However for TGFBI, there was only one tag belonging to MPSS tag class 1 which showed marginal over-expression in GBM compared to normal tissues (17 tpm vs 0 tpm, FDR 0.23). To evaluate the true expression pattern of TGFBI and SOX4 in GBM and normal brain tissues, we performed quantitative RT-PCR on a panel of 19 individual brain tumor samples and 9 individual normal brain tissues and showed that TGFBI and SOX4 RNA expression are significantly higher (P values of 3.18E-03 and 2.01E-03 respectively, T-test, two-tailed distribution, unequal variance) in GBM tissues compared to normal brain tissues (Figure 4). For TGFBI, there were two clusters, one with higher TGFBI expression and another with TGFBI levels similar to that of normal tissues. For SOX4, the majority of the GBM tissues expressed higher levels compared to normal tissues, with three exhibiting extremely high expression (41 to 108 times higher than the median of the expression in all tissues, data not shown).

**Figure 5:**
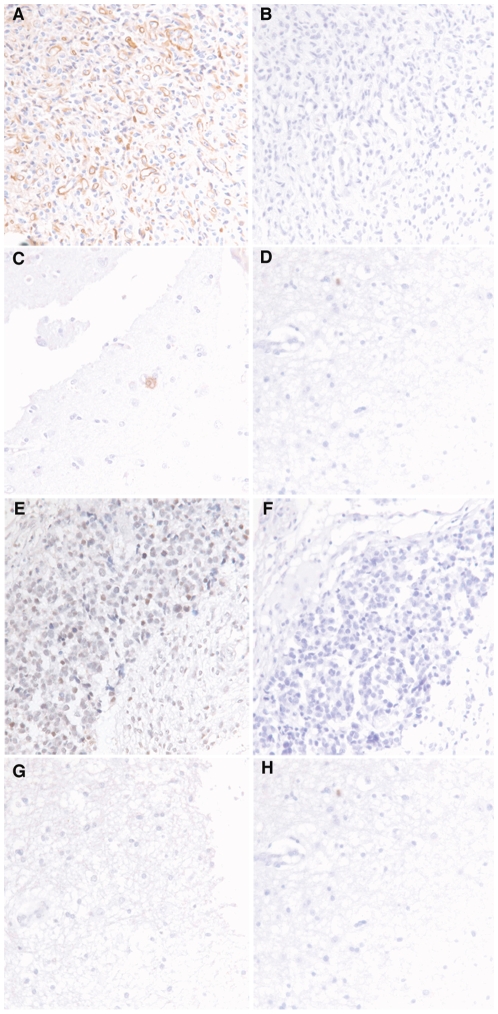
Examples of IHC staining of GBM and normal brain tissue samples. A: TGFBI (GENE ID:7045) antibody staining of GBM tissue; B: IgG staining of GBM tissue; C: TGFBI (GENE ID:7045) antibody staining of normal brain tissue; D: IgG staining of normal brain tissue. E: SOX4 antibody staining of GBM tissue; F: IgG staining of GBM tissue; G: SOX4 antibody staining of normal brain tissue; H: IgG staining of normal brain tissue. Please see Table 1 for data summary of the entire tissue array.

To further analyze whether the protein products of these two genes are over expressed in GBM, we performed immunohistochemistry analysis of SOX4 and TGFBI in 60 GBM tissues and three normal brain tissues (TMA CS17-01-004 from Cyberdi Inc.). For TGFBI, we observed strong tumor specific immunoreactivities in most GBM samples with extra cellular staining pattern mainly in tumor cells and in malignant vasculature endothelial cells but negative staining in normal brain tissues (Figure 5). For SOX4, we observed positive immunoreactivities in GBM samples with nucleus staining pattern in tumor cells but negative staining in normal brain tissues (Figure 5). Examples of IHC staining results are shown in Figure 5 and a summary table is shown in Table 2. Statistical analysis using Fisher's exact test indicates that TGFBI (GENE ID:7045) show statistically significant protein expression differences between GBM and normal tissues (P = .0037). However, the difference in protein expression between GBM and normal tissues for SOX4, although at 60% positive rate in GBM vs. 0% positive rate for normal tissues, was not statistically significant by the Fisher's exact test (P = 0.083) (Table 2).

**Table 2:** Summaries of IHC staining of TGFBI and SOX4 on GBM and normal brain tissues.

Pathology	TGFB1			SOX4		
	Negative	Positive	Positive Rate (%)	Negative	Positive	Positive Rate (%)
Normal	3	0	0	3	0	0
Glioblastoma	3	27	90	12	18	60
Fisher's Exact Test			.0037*			.083
*P<0.05						

**Figure 6:**
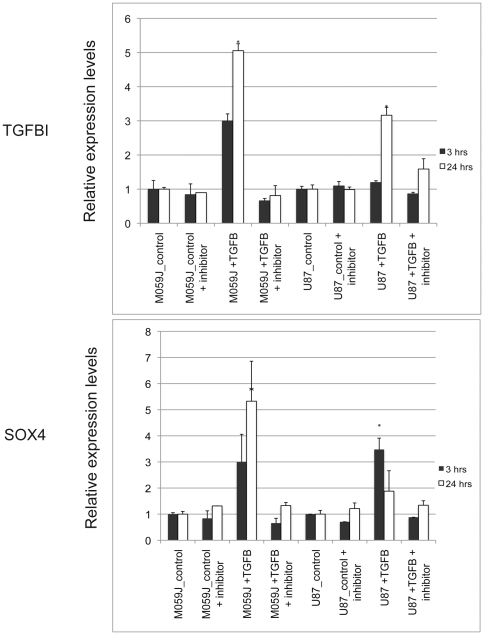
RT-PCR results showing the expression changes of TGFBI (GENE ID:7045) and SOX4 in response to TGF−β stimulation with and without inhibition of TGF−β receptor I (TβRI) kinase in two GBM cell lines, M059J and U87MG. Y-axis indicates relative expression levels with standard deviation indicated on top of the each bar. X-axis indicates cell types and treatment conditions (vehicle control, vehicle control plus TβRI kinase inhibitor LY210976, TGF−β stimulation, TGF−β stimulation in the presence of TβRI kinase inhibitor LY210976). The expression levels of TGFBI (GENE ID:7045) and SOX4 were measured at two time points 3 hours and 24 hours (* indicates P<0.05, T test, two-tailed distribution, unequal variance).

To determine whether TGF beta 1 indeed acts on GBM cells to change the expression of SOX4 and TGFBI (GENE ID:7045) and to see whether the change was acted through TGF–β receptor I kinase (TβRI), we stimulated two different GBM cell lines, U87MG and M059J, with TGF−β and then inhibited the TGF−β pathway with a specific inhibitor of the TGF–β receptor I kinase (TβRI). We detected increased expression of both TGFBI (GENE ID:7045) and SOX4 after addition of 100 pM of TGF−β (R&D Systems) at 3 hour and 24 hour time points (Figure 6). We also observed that M059J cells were more sensitive to TGF−β treatment than U87 cells, responding with a higher amplitude than in U87 cells. We also noticed that the response to TGF−β was higher at the 24-hour than at the 3-hour time point for TGFBI (GENE ID:7045) for both M059J and U87 cells. For SOX4, responses were higher at the 24-hour than the 3-hour time point in M059J cells and reversed, higher at the 3-hour than the 24-hour time point, in U87 cells (Figure 6). This suggests that individual GBM cell lines respond differently in amplitude and in time to TGF−β treatment. Adding the specific TβRI inhibitor LY2109761 (Calbiochem) reversed the effects of TGF−β stimulation resulting in decreased expression of TGFBI (GENE ID:7045) and SOX4 (Figure 6). These data indicate that SOX4 and TGFBI (GENE ID:7045) are TGF –β responsive genes.

## Discussion

We applied MPSS technology to compare the expression profiling of a pool of five normal brain tissues to a pool of five GBM tissues. The pooled sample strategy was used because of the cost of doing MPSS at the time. The pooled strategy for MPSS analysis had been validated previously in other studies. For example, Grigoriadis et al. used a pool sample strategy for MPSS analysis in which they identified 6,553 differentially expressed genes between the pool of normal luminal cells and that of primary tumors substantially enriched for epithelial cells [31]. Our dataset should prove to be a useful resource for the research community, however there are several limitations in our approach which should be acknowledged. By pooling samples, biological variance (e.g. tumor heterogeneity or true normal individual variance) could not be measured. Although we have confirmed differential expression of several genes by RT-PCR (Figure 2 and Table S5), we cannot exclude the possibility that our MPSS data might over or under estimate differential expression for other genes showing biological variance. Furthermore, although we included technical replicates in the MPSS analysis (replicate two stepper and replicate 3 stepper sequencing runs), variation for the sample preparation step was not measured. Finally, there may be confounding effects related to a small sample size in a relatively heterogeneous disease. Our five GBM samples came from patients with primary GBM with typical clinical characteristics (mean age  = 62, length of survival <15 months) suggesting a non-proneural classification [32]. The non-tumor brain samples consisted of histologically-normal temporal lobe white matter obtained from patients undergoing surgery for epilepsy. While matched for sex (M:F ratio 3∶2), the average age of the epilepsy patients was younger (mean age  = 25 years). As demonstrated in our analysis, differential gene expression based on this MPSS strategy should be confirmed by RT-PCR in a larger independent sample set.

In this study, we identified and confirmed that FOXM1 is over expressed in GBM comparing to normal brain tissues. Our data is consistent with previous observation that FOXM1 is over expressed in GBM and its protein expression levels are inversely correlated with patient survival [33]. Over expression and knock-down studies of FOXM1 suggested that FOXM1 confers GBM tumorigenicity [33] and increases tumor invasion by enhancing matrix metalloproteinase-2 expression [34]. We confirmed that both members of the mammalian chitinase-like proteins were over expressed in GBM comparing to normal brain tissues (Figure 2). YKL39 differs from YKL40: while YKL40 has chitinase activity, YKL-39 was predicted to lack chitinase activity as it the active site glutamate [18]. Furthermore, In contrast to YKL-40, YKL-39 is not a glycoprotein and does not bind to heparin [18]. Western blot and ELISA analysis suggested that YKL-40 serum levels were significantly increased in many GBM patients and that serum YKL-40 levels correlate with tumor grade [16]. It will be interesting to see whether YKL-39 can be used as serum biomarker for GBM to increase specificity and sensitivity of cancer diagnosis and stratifications.

We identified decreased expression of NRGN (neurogranin) and L1CAM (L1 cell adhesion molecule) in GBM tissues compared to normal brain tissues (Figure 2). Neurogranin, a calmodulin (CaM)-binding protein kinase C (PKC) substrate, is a brain and neuron-specific gene [35]. It regulates the availability of Ca(2+)/CaM complex and modulates the homeostasis of intracellular calcium in neurons and it may be involved in selective vulnerability of neurons to oxidative insults in the CNS [36]. L1CAM is a cell adhesion molecule that plays an important role in nervous system development, including neuronal migration and differentiation [37]. L1CAM has been shown to be involved in C6 rat glioma cell migration via its imunoglobulin C2-like domain [38], however, the expression of L1CAM is lower in glioma when compared to a neuroblastoma cell line [38]. While not surprising to find decreased neuronal genes in GBM, the role of decreased expression of NRGN and L1CAM may have important functional consequences which have not been previously studied in GBM.

Visual inspection of the canonical TGF–β pathway overlaid with MPSS derived gene expression changes revealed several genes in the canonical TGF–β pathway up-regulated in GBM compared to normal tissues including TGF–β 1, and its effectors SMAD2 and SMAD7. TGF–β ligands bind to heterotetrameric complexes of type I and type II receptors of TGF–β (TGF–βRII and TGF–βRI), activate TGF–βRI to phosphorylate SMAD2 and/or SMAD3. Phophorylated SMAD2/3 then disassociate from SARA (SMAD anchor for receptor activation), allowing SMAD2/3 to bind to SMAD4. The resulting complex is then translocated to the nucleus and activates gene transcription [39], [40]. Our data suggest that the SMAD mediated TGF–β pathway is activated in GBM. Previously, it was shown that TGF–β expression was increased in GBM compared to normal brain tissues and its expression may be related to malignancy of glioma [41], [42]. That observation is consistent with our data. The role of TGF-β in GBM is complex and not fully elucidated. TGF–β has been shown to be involved in multiple processes in GBM including excessive proliferation, infiltrative growth, angiogenesis and suppression of anti-tumor immune surveillance [43]. A central question remains as to how advanced brain tumors such as GBM lose the growth suppressive effects of TGF–β but retain TGF–β mediated proliferative and invasive properties [44].

We furthered the analysis of the TGF–β signaling network in GBM by integrated analysis of TGF–β regulated genes and differentially expressed genes between GBM and normal brain tissues. We built an expanded TGF–β signaling network and overlaid to it the expression changes found in GBM compared to normal brain tissues. In additional to the canonical SMAD mediated TGF–β signaling module, we identified two interesting modules centered on SOX4 and TGFBI (GENE ID:7045). We further showed that SOX4 and TGFBI (GENE ID:7045) are over expressed at both the mRNA and protein levels in GBM compared to normal brain tissues by quantitative RT-PCR and IHC staining (Figure 4-5). Finally, we demonstrated that both SOX4 and TGFBI (GENE ID:7045) are responsive to TGF–β stimulation acting through TβRI as adding TβRI inhibitor LY2109761 reversed the stimulative effects of TGF−β on TGFBI (GENE ID:7045) and SOX4 expression (Figure 6).

Our integrative analysis identified that TGF−β signaling through SOX4 or TGFBI appears to be activated in GBM compared with normal brain tissues. In the RT-PCR data, we noticed that SOX4 and TGFBI appear to be almost mutually exclusive in the tumor samples, suggesting a possibility that GBM may achieve activation of the non-canonical TGF-β through either SOX4 or TGFBI. Further experiments would be necessary to confirm this possibility. Recently, Ikushima et al. demonstrate that TGF-beta induces the expression of SOX2, a stemness gene, in glioma-initiating cells (GICs) and that the induction was mediated by SOX4 [45]. They further showed that inhibition of TGF-beta signaling drastically decreased the tumorigenicity of GICs by promoting their differentiation, and that these effects were attenuated in GICs transduced with SOX2 or SOX4. Taken together with our findings, this study supports the potentially important role of TGF-beta signaling through the SOX4 protein in gliomas.

Our analysis expanded our knowledge of the TGF–β signaling network and suggested that TGF–β signaling through SOX4 might be an alternative non-SMAD mediated TGF–β signaling pathway. SOX4 is a transcriptional activator that may play a role in central nerve system development [46]. SOX4 is a protein with diverse functions and has been implicated in multiple cancers [47] [48] [49] [50]. For example, it can regulate beta-catenin/T-cell factor activity and proliferation of colon carcinoma cells [47]. De Bont showed that SOX4 is over expressed by about 11 fold in medulloblastoma compared with ependymoma and normal cerebellum [48]. Pramoonjago et al. showed that SOX4 is one of the most up regulated genes in adenoid cystic carcinoma (ACC) compared to non-neoplastic tissues. They further demonstrated that RNA interference (RNAi)-mediated RNA silencing of SOX4 increases cell apoptosis and reduces cell survival in the ACC-derived cell line ACC3, suggesting that Sox4 could contribute to the malignant phenotype of ACC cells by promoting cell survival [49]. Recently, SOX4 was shown to bind to the promoter of EGFR and transcriptionally activates EGFR [27]. Other growth factors targeted by SOX4 include FGFRL1, and IGF2R. Recently, both SOX4 and tenascin C were shown to enhance metastasis of breast cancer cells to the lung [50].

We identified a TGFBI (GENE ID:7045) module containing extracellular matrix proteins that are over expressed in GBM compared to normal brain tissues. These genes include collagens (e.g. COL1A2, COL1A1, COL2A1, COL4A2), MMP2 (matrix metalloproteinase 2), SPARC (secreted protein, acidic and rich in cysteine) and fibronectin (FN) (Figure 3). SPARC has been shown to promote GBM invasion *in intro* [51] and MMP2 expression was increased in GBMs [52]. TGFBI (GENE ID:7045) itself is an extracellular matrix protein that promotes metastasis in colon cancer by enhancing cell extravasation [53].

We compared our MPSS data with the Cancer Genome Atlas (TCGA) data for GBM (http://cancergenome.nih.gov/dataportal/) [54]. We believe that our dataset will be a useful resource to complement the TCGA expression data, which has been generated using two major array platforms—Affymetrix and Agilent. For many lowly expressed genes, the MPSS technology seems to have better sensitivity and reliability in detecting changes in expression. For example, evaluating the Broad-MIT's U133A dataset (TCGA) of 173 GBM samples for genes that we identified as up regulated by MPSS, about 30% are expressed in the lower 20% percentile of the array raw intensity (data not shown) and these are known not to be reliably detected as their expression levels are close to background. In addition, MPSS offer the advantage of identifying and comparing different RNA isoforms for the same gene. Although we did not discuss in detail the RNA isoforms in our dataset, our raw data (Table S1) will be a useful resource for investigators interested in RNA isoforms, especially the differentially polyadenylated RNA isoforms that MPSS has a strength in identifying.

In summary, we have identified non-SMAD mediated TGF−β signaling pathways acting through SOX4 and TGFBI (GENE ID:7045) in GBM. These pathways warrant further investigation and should be considered, in addition to the canonical SMAD mediated pathway, in the development of new therapeutic strategies targeting TGF−β signaling in GBM.

## Materials and Methods

### Ethics Statement

All patients signed a written informed consent and the data and samples were analyzed anonymously. The present study was approved by the Institutional Review Boards of the University of Iowa and Swedish Medical Center.

### Tissue samples and cell lines

Histologically-confirmed GBM and histologically normal non-tumor brain specimens (temporal lobe white matter from epilepsy resections) were obtained from the University of Iowa Hospital. All patients gave informed consent prior to collection of specimens according to institutional guidelines. An equal amount of RNA from five histologically-normal non-tumor white matter specimens were pooled and used for MPSS analysis. The same was done for five histologically-confirmed GBM samples. The samples were sequenced as normal pool and cancer pool (not barcoded or multiplexed).

Glioblastoma cell line U87MG and M059J were obtained from ATCC (http://www.atcc.org/). Both cell lines were passaged in the laboratory for fewer than 6 months after resuscitation. The cells were authenticated by cytogenetic analysis and typing of isozymes by ATCC. Cells were maintained at 37°C in a 5% CO_2_-95% air atmosphere in a media consisting of DMEM, 10% fetal bovine serum, and 100 units/100 mg per ml penicillin/streptomycin.

Total RNA was extracted using Trizol (Invitrogen, Carlsbad, CA) and additional purification performed using RNeasy MinElute Cleanup kit (Qiagen, Valencia, CA) before quality assessment with the Agilent Bioanalyzer (Palo Alto, CA).

### MPSS analysis

A pool of five normal brain tissues and a pool of five GBM tissues were analyzed by the “signature cloning” variety of the MPSS technology. In brief, RNAs were captured with microbeads containing olido dT and cDNA synthesized on beads. cDNA were then digested by *DpnII* restriction enzyme and the Dpn-II-to-polyA-fragments were captured on beads. An adaptor with *MmeI* recognition site was ligated to the 5′-ends of the Dpn-II-to-polyA-fragments, followed by *MmeI* digestion that cuts 21–22 bases downstream. This 21–22 base signature from each transcript was subsequently cloned using adaptors and loaded to microbeads for sequencing. The libraries were constructed and sequenced at Lynx Therapeutics, Inc (now Illumina Inc.) (Hayward, CA). For sequencing the MPSS tags, sequencing runs were done by using two different sequencing reactions that results in sequence determination that is offset by two bases (2-step) or three bases (3-step) as described previously [11]. }. These sequencing reactions are hereafter called steppers i.e. “2-stepper” or “3-stepper”. Four technical replicates were conducted for individual sequencing runs. After 2-stepper or three-stepper sequencing determination, the counts for a given tag were summed and averaged by the two-stepper or the three-stepper sequencing reactions. The sequencing stepper with higher count average was selected to represent the tag. Replicated runs of the chosen stepper sequencing were averaged as the final representation for the tag. The expression data were normalized to per million, which is expressed as tags per million (tpm).

### Identification of differentially expressed genes (DEGs)

The following method was applied to compute the FDR using two sets of two technical replicates: for the normal or GBM sample, two sets of tag counts from two independent sequencing runs of the same sample. First, to compute a distribution of the complete null hypothesis that the tag counts were not different between normal and GBM, we applied the Z-test in powerSAGE [15] to 1) two normal and 2) two GBM technical replicates (i.e. normal vs. normal and GBM vs. GBM), resulting in two sets of p-values from the two comparisons. Second, we then computed Z values for the two sets of p-values as 1-Ncdf^−1^(P) where Ncdf^−1^ is the inverse function of the standard normal cumulative density function, and P is a p-value for each tag from the Z-test. Third, we combined the Z values for the two sets of p-values to generate an empirical distribution of the null hypothesis. Fourth, we computed the Z values for the normal vs. GBM comparison by applying the Z-test to the mean tag counts of normal and GBM technical replicates and then applying 1-Ncdf^−1^(P) to the resulting p-values from the Z-test. Fifth, for a Z value (*Z_i_*) for each tag (*t_i_*) from the previous step, the expected fraction of false positives under the complete null hypothesis was estimated as the fraction of tags with Z > *Z_i_* in the empirical null hypothesis distribution. Sixth, for each tag, FDR was computed as the expected fraction of false positives multiplied by two times the number of tags with p-values>0.5 divided by the total number of tags, according to Storey's method [55], [56]. Finally, the differentially expressed genes were selected as the tags with FDR<0.1. To identify differential expression for genes with multiple tags, where different tags could represent different isoforms of the same gene, we accounted for and analyzed each of the individual tags. As long as one of the tags of a gene showed differentially expression, we considered the gene as differentially expressed.

### Gene ontology and pathway enrichment analysis

For gene ontology analysis, we used gominer (http://discover.nci.nih.gov/gominer/). The background list was all transcripts identified by MPSS. GO biological processes at level 3 were used for gene ontology categories. GO terms with FDR <0.05 were considered significantly enriched. For GSEA analysis (http://www.broad.mit.edu/gsea/index.jsp), we used the molecular signature database C2, which contains 1892 curated gene sets that are collected from various sources including online pathway databases and knowledge of domain experts. Permutations of the gene sets 1000 times were performed to calculate the P values and FDR q values. Other basic parameters were set as default except the metric for ranking genes was set to ratio of classes; minimum size of enriched sets, 15.

### Real-time quantitative PCR

Purified RNA (1 µg) was reverse transcribed using random primers (Applied Biosystems). The resulting cDNA is diluted 25 fold and used as template. Real-time PCR is performed using Assay on Demand gene expression reagents (Applied Biosystems) on ABI PRISM 7900 HT Sequence Detection System under default conditions: 95°C for 10 min, and 40 cycles of 95°C for 15 s and 60°C for 1 min. The expression of human GUS (beta glucuronidase) was used as endogenous control and comparative C_t_ method was used for quantification of the transcripts. Measurement of ΔC_t_ was performed in triplicate.

### Imunohistochemistry

Rabbit polyclonal anti-human TGFBI (GENE ID:7045) (Transforming Growth Factor-Beta Induced, 68-KD;) antibody (Proteintech Group, Cat# 10188-1-AP) and Rabbit polyclonal anti-human SOX4 antibody (Abcam, Cat# 52043) were used for IHC staining. For control, Mouse IgG isotype control antibody 250 ug/ml (BD, Cat# 550878) and Rabbit IgG isotype control antibody 5 mg/ml (Southern Biotech, Cat# 0111-01) were used. Brain tissue array (Lot ID: CS17-01-004) from Cybrdi, Inc. was used (http://cybrdi.com/viewproduct.php?id=305). Tissue arrays were formalin fixed, paraffin embedded (PPFE) array slides. Each tissue array contained 60 paired tissue cores from 30 different GBM tumor samples and three tissue cores from normal brain. The tissue array core diameter was 1.5 mm with a core thickness of 5 µM.

We used the IHC services provided by Cybrdi, Inc. including antibody optimization, IHC staining, pathological reading and scoring by experienced pathologists. Primary antibodies for TGFBI (GENE ID:7045) and SOX4 were diluted at 1∶100, 1∶25 respectively for IHC. Secondary antibody was used at 1∶200 dilution. For isotype control antibodies, rabbit IgG was used at 1∶25 and murine IgG was used at 1∶10. The scoring criteria contain two parameters, percentage of positive cell population and staining intensities. For percentage of positive cell population, the categories are: 0 = 0% of the cell population is positive; 1 = 1 to 25% of the cell population is positive: 2 = 26 to 50% of the cell population is positive: 3 = 51 to 75% of the cell population is positive: 4 = 76 to 100% of the cell population is positive. The staining intensities were scored as: -  =  Negative staining; +  =  Weak staining intensity; ++  =  Medium staining intensity; +++  =  Strong staining intensity.

### TGF−β treatments

Human Glioma cell lines (U87MG and M059J) were serum deprived for 24 hrs prior to treatment of TGF−β 1 (Gene ID: 7040) (100 pM, R&D Systems) and/or TGF−β 1 Receptor Kinase Inhibitor (LY-364947, 2 uM, Cal biochem) for 3 hr or 24 hrs in serum-free media.

## Supporting Information

Table S1 All MPSS tags identified and their expression data in the normal brain tissues and the GBM tissues.
(5.78 MB XLS)

Table S2 Known genes belonging to MPSS class 1 to 5.
(4.27 MB XLS)

Table S3 Up-regulated genes in GBM tissues compared with normal tissues.
(0.46 MB XLS)

Table S4 Down-regulated genes in in GBM tissues compared with normal tissues.
(0.48 MB XLS)

Table S5 Selected genes for PCR.
(0.03 MB XLS)

Table S6 Enriched gene sets in GBM tissues.
(0.03 MB XLS)

Table S7 Enriched gene sets in normal brain tissues.
(0.02 MB XLS)

Table S8 TGF beta related genes differentially expressed between GBM and normal tissues.
(0.09 MB XLS)

Figure S1 The canonical TGF−β pathway in KEGG with overlaid expression changes of GBM tissues to normal brain tissues. Red color indicates up-regulated and blue color indicates down regulated genes. Yellow color indicates no significant change in expression was observed.
(2.94 MB TIF)
