# Supplementary figures and images for: Massively Parallel Signature Sequencing and Bioinformatics Analysis Identifies Up-Regulation of TGFBI and SOX4 in Human Glioblastoma
Source: PLoS One. 2010 Apr 19;5(4):e10210. doi: 10.1371/journal.pone.0010210 (PMC2856677; doi:10.1371/journal.pone.0010210)

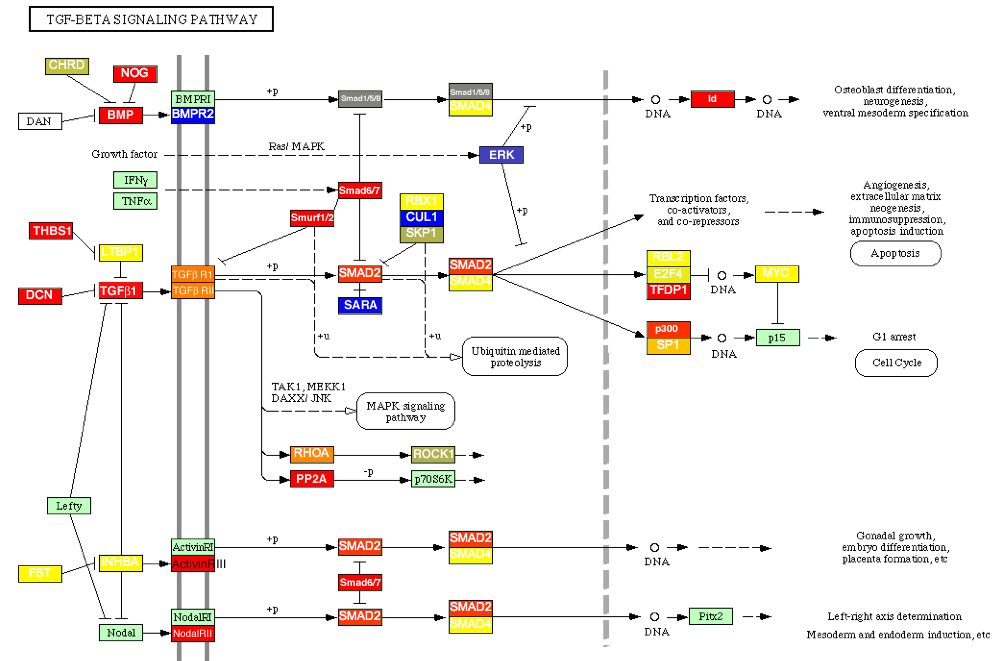

Supplement: Figure S1 — The canonical TGF−β pathway in KEGG with overlaid expression changes of GBM tissues to normal brain tissues. Red color indicates up-regulated and blue color indicates down regulated genes. Yellow color indicates no significant change in expression was observed. (2.94 MB TIF) [file pone.0010210.s009.tif]
